# Effect of excessive gestational weight gain before and after 28 weeks on trial of labor after cesarean stratified by pre-pregnancy body mass index: a retrospective cohort study

**DOI:** 10.3389/fmed.2023.1157967

**Published:** 2023-08-10

**Authors:** Guangpu Liu, Jingya Zhang, Chaofan Zhou, Huixin Zhang, Haoran Shen

**Affiliations:** ^1^Department of Obstetrics and Gynecology, The Fourth Hospital of Hebei Medical University, Shijiazhuang, Hebei, China; ^2^Department of Neurology, Children’s Hospital of Hebei Province, Shijiazhuang, Hebei, China; ^3^Department of Gynecology, Obstetrics and Gynecology Hospital of Fudan University, Shanghai, China

**Keywords:** trial of labor, weight gain, cesarean section, body mass index, pregnancy

## Abstract

This study aimed to assess the effect of excessive gestational weight gain (GWG) before and after 28 weeks on the mode of delivery in women who attempted a trial of labor after cesarean (TOLAC), stratified by pre-pregnancy BMI. A retrospective analysis of the outcomes of eligible women who attempted trial of labor after cesarean (TOLAC) in a Chinese hospital from January 2016 to October 2022 was performed. GWG before and after 28 weeks was categorized as ‘excessive’ or ‘non-excessive’ based on the guideline of Institute of Medicine (IOM). Multivariable logistic regression analyses were used to estimate the effect of excessive GWG before and after 28 weeks on mode of delivery in women who underwent TOLAC, stratified by pre-pregnancy BMI. Of the 512 women who underwent term trial of labor, 71.1% achieved a vaginal birth. No correlation was found between excessive GWG before 28 weeks and the rate of vaginal birth after cesarean (VBAC). Among women with or without excessive GWG before 28 weeks, excessive GWG after 28 weeks was significantly associated with a reduced rate of VBAC. When stratified by pre-pregnancy BMI, women who had excessive gestational weight gain after 28 weeks gestation had lower rates of VBAC than those who did not, regardless of being underweight, normal or overweight (aOR 0.23, 95% CI 0.06–0.88; aOR 0.42, 95% CI 0.25, 0.70; and aOR 0.12, 95% CI 0.04–0.36; respectively). Excessive weight gain after 28 weeks of pregnancy was related to decreased rates of VBAC, irrespective of pre-pregnancy weight status and weight gain before 28 weeks.

## 1. Introduction

Women who have had a prior cesarean section can choose either vaginal birth after cesarean section (VBAC) or repeat cesarean section (RCS) for their subsequent deliveries. However, globally, most women with a previous cesarean opt for a repeat cesarean. The rate of trial of labor after cesarean (TOLAC) differs across countries, with lower rates in China (10%) and the US (13%) and a higher rate in Germany (36.0–49.8%) (1–3). Although successful TOLAC is associated with lower overall morbidity rates than RCS (4), concerns about its safety and associated liability continues to limit its availability (5–8).

The factors that impact VBAC rates include onset of labor, previous vaginal birth or VBAC, multiple cesarean sections, body mass index (BMI), and interpregnancy interval (9–12). Gestational weight gain (GWG) is a potential risk factor for the global obesity crisis, and unlike most of the other factors, it can be modified during pregnancy (13, 14). Excessive GWG can lead to complications of a kind that cesarean section (CS), preeclampsia, gestational diabetes, large for gestational age babies (LGA), and macrosomia (15–17). Only a handful of studies have examined the link between GWG and VBAC, and these studies have yielded inconsistent results. Two studies from the USA and New Zealand indicated that excessive GWG could lower the chances of vaginal birth (18, 19), while another study from the USA detected no variation in TOLAC success rates with excessive GWG (20).

The Institute of Medicine (IOM) guideline proposed fitting GWG classified by pre-pregnancy BMI in 2009 (21). Maternal metabolic changes and GWG differ throughout pregnancy (22); therefore, some studies specifically examined the association between excessive GWG during the first or second trimester and pregnancy complications (16, 23, 24). However, the association between excessive GWG at different stages of pregnancy and VBAC remains unclear. This study aims to explore the relationship between weight gain before and after 28 weeks and VBAC stratified by pre-pregnancy BMI.

## 2. Participants and methods

### 2.1. Study setting

This was a retrospective study performed at the Fourth affiliated Hospital of Hebei Medical University, which is a comprehensive regional medical center located in northeast China that performs approximately 1,800 deliveries per year. Before week 8 of gestation, a specific electronic file was created for each pregnant woman at their first antenatal visit. They then received regular follow-up visits every 2–4 weeks until delivery. The study was approved by the Ethics Committee of the Fourth affiliated Hospital of Hebei medical university (2022KS010).

### 2.2. Eligibility criteria

The study population consisted of women who registered at their first prenatal visit before week 8 of gestation and attempted TOLAC and delivered between January 1, 2016 and October 31, 2022. We excluded women with malpresentation in the current pregnancy, previous uterine scar other than low transverse incision scar, multiple gestation, more than one prior cesarean delivery, placenta previa, delivery before week 37 (37 0/7 weeks), and incomplete information. We also omitted obese women (BMI > 30 kg/m2) from the study, as this group had an insufficient sample size for representation.

### 2.3. Data collection

For the analysis, the clinical characteristics of women attempting TOLAC were obtained from electronic files, including maternal age, maternal height, labor induction, gestational age at delivery, maternal hypertension or diabetes in pregnancy, previous vaginal birth or VBAC, pre-pregnancy weight, maternal weight at 28 weeks, maternal weight at delivery, and mode of delivery. Meanwhile, the weight of each participant was recorded at each visit; they wore light clothing without shoes during the measurement.

### 2.4. Determination of gestational weight gain

During the first antenatal care visit, each participant reported their pre-pregnancy weight. Maternal weight at 28 weeks was determined as the latest weight measured within 1 week around 28 weeks. Maternal weight at delivery was that measured within 2 days before the delivery date. By dividing the self-reported pre-pregnancy weight (25, 26) (in kilograms) of each participant by their height squared (in meters), the pre-pregnancy BMI was calculated (27). GWG was calculated as follows: GWG before 28 weeks = maternal weight at 28 weeks - pre-pregnancy weight; GWG after 28 weeks = maternal weight at delivery - maternal weight at 28 weeks.

We applied the recommendation of IOM for weight gain during pregnancy based on pre-pregnancy BMI (underweight: < 18.5 kg/m^2^, normal: 18.5–24.9 kg/m^2^, overweight: 25.0–29.9 kg/m^2^) (21) (Table 1) to calculate the suitable range of gestational weight gain before and after 28 weeks (Table 2). Using this standard, we categorized women as having ‘excessive GWG’ if they exceeded the recommended upper limit for their BMI group, and ‘non-excessive GWG’ if they gained weight within or below the recommended range.

**Table 1:** Gestational weight gain recommended by the IOM guideline (21).

Pre-pregnancy weight category	Pre-pregnancy body index (kg/m^2^)	First trimester GWG Range (kg)	Second and Third trimester rate of GWG (kg/week)
Underweight	<18.5	0.5–2.0	0.51 (0.44–0.58)
Normal	18.5–24.9	0.5–2.0	0.42 (0.35–0.50)
Overweight	25.0–29.9	0.5–2.0	0.28 (0.23–0.33)

GWG, gestational weight gain; IOM, Institute of Medicine.

**Table 2:** Gestational weight gain of included pregnant women according to IOM standard (21).

Pre-pregnancy weight category	Pre-pregnancy body index (kg/m^2^)	Range of GWG before 28 weeks (kg)	Range of GWG after 28 weeks (kg)
Underweight	<18.5	6.66 ~ 10.12 (0.5 + 14 × 0.44 ~ 2 + 14 × 0.58)	(w-28) × (0.44 ~ 0.58)
Normal	18.5–24.9	5.04 ~ 8.72 (0.5 + 14 × 0.35 ~ 2 + 14 × 0.5)	(w-28) × (0.35 ~ 0.5)
Overweight	25.0–29.9	3.08 ~ 7.18 (0.5 + 14 × 0.23 ~ 2 + 14 × 0.33)	(w-28) × (0.23 ~ 0.33)

GWG, gestational weight gain; IOM, Institute of Medicine.

### 2.5. Data analysis

We evaluated participant characteristics across GWG before and after 28 weeks by frequency (percentage), and used the Chi-squared or Fisher exact tests to compare the distribution of differences. We analyzed continuous variables using the Student’s t test. Predictors of VBAC success were evaluated using univariate logistic regression analyses. Then, we adjusted for potential confounders that were statistically significant in the univariate analysis (those with *p* < 0.05) and generated multivariable logistic regression models to investigate the association between gestational weight gain before and after 28 weeks and VBAC.

To understand how GWG before and after 28 weeks affects VBAC, a stratified analysis was performed to examine the relation between GWG after 28 weeks and the rate of VBAC in groups of GWG before 28 weeks (classified into excessive and non-excessive). The non-excessive GWG group was used as the reference group.

**Figure 1:**
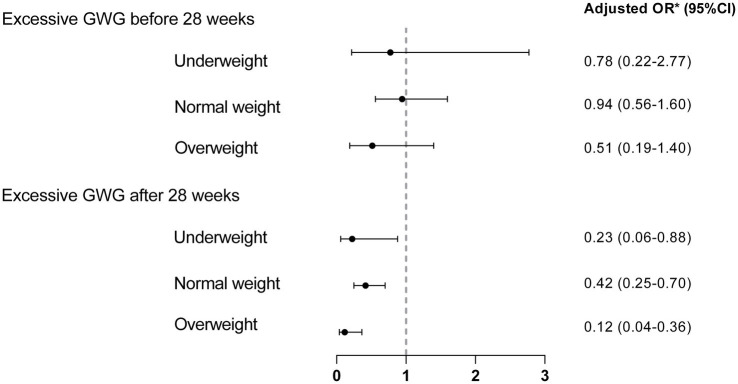
Association between excessive GWG before and after 28 weeks and VBAC stratified by pre-pregnancy BMI. Non-excessive GWG was used as the reference group. *Adjusted for induction of labor, hypertension in pregnancy, previous vaginal birth or VBAC.

We also generated multivariable logistic regression models for the three subgroups (underweight, normal weight, and overweight) to further explore the relation between excessive gestational weight gain before and after 28 weeks and VBAC rate among women with different pre-pregnancy BMI categories, adjusting for the same confounders except pre-pregnancy BMI. The adjusted OR with 95% CI are shown in Figure 1. SPSS version 19.0 software was used for all statistical analyses, and GraphPad Prism version 6.0 was used to draw the figure. A two-tailed *p* < 0.05 indicated statistical significance for analyses.

## 3. Results

We examined 552 women who attempted TOLAC for eligibility during the study period. Thirty-one women who did not fulfil the eligibility criteria and 9 women were obese were excluded. In total, 512 (92.7%) women were included in the analysis. Nearly half of the 512 women who met the inclusion criteria exceeded the IOM recommendations for GWG after 28 weeks (45.5%), while 33.8% had excessive GWG before 28 weeks. The demographics of the participants are summarized in Table 3, stratified by GWG before and after 28 weeks. GWG before 28 weeks was not significantly influenced by hypertension or diabetes in pregnancy, maternal age, previous vaginal birth or VBAC, maternal height, gestational age at delivery, pre-pregnancy BMI, or induction of labor. However, GWG after 28 weeks was associated with pre-pregnancy BMI, hypertension in pregnancy, diabetes in pregnancy, and induction of labor. Pre-pregnancy overweight women had a higher likelihood of exceeding the IOM recommendations after 28 weeks (*p* < 0.001). Hypertension in pregnancy and induction of labor was more likely to occur in women with excessive GWG after 28 weeks (*p* = 0.048, p < 0.001, respectively), while women with diabetes in pregnancy tended not to exceed the IOM recommendations after 28 weeks (*p* = 0.004).

**Table 3:** Participant characteristics by gestational weight gain group.

Characteristics	GWG before 28 weeks			After 28 weeks		
	Not excessive (*n* = 339, 66.2%)	Excessive (*n* = 173. 33.8%)	*p*-value	Not excessive (*n* = 279, 54.5%)	Excessive (*n* = 233, 45.5%)	*P*-value
Age (y)			0.642			0.540
<35	261 (77.0)	130 (75.1)		216 (77.4)	175 (75.1)	
≥35	78 (23.0)	43 (24.9)		63 (22.6)	58 (24.9)	
Maternal height (cm)	162.36 ± 5.06	162.60 ± 5.06	0.610	162.18 ± 5.23	162.76 ± 4.83	0.193
Pre-pregnancy BMI (kg/m^2^)			0.929			<0.001
<18.5	35 (10.3)	16 (9.2)		37 (13.3)	14 (6.0)	
18.5–24.9	250 (73.7)	129 (74.6)		218(78.1)	161 (69.1)	
25–29.9	54 (15.9)	28 (16.2)		24 (8.6)	58 (24.9)	
Gestational age of delivery			0.187			0.795
<40 weeks	237 (69.9)	111 (64.2)		191 (68.5)	157 (67.4)	
≥40 weeks	102 (30.1)	62 (35.8)		88 (31.5)	76 (32.6)	
Previous vaginal birth or VBAC			0.225			0.242
Yes	28 (8.3)	20 (11.6)		30 (10.8)	18 (7.7)	
No	311 (91.7)	153 (88.4)		249 (89.2)	215 (92.3)	
Hypertension in pregnancy			0.104			0.048
Yes	36 (10.6)	27 (15.6)		27 (9.7)	36 (15.5)	
No	303 (89.4)	146 (84.4)		252 (90.3)	197 (84.5)	
Diabetes in pregnancy			0.509			0.004
Yes	42 (12.4)	18 (10.4)		43 (15.4)	17 (7.3)	
No	297 (87.6)	155 (89.6)		236 (84.6)	216 (92.7)	
Induction of labor			0.292			<0.001
Yes	70 (20.6)	29 (16.8)		37 (13.3)	62 (26.6)	
No	269 (79.4)	144 (83.2)		242 (86.7)	171 (73.4)	

GWG, gestational week gain. BMI, body mass index. VBAC, vaginal birth after cesarean section.

Among all women who attempted TOLAC, vaginal delivery occurred in 71.1%. The timing of weight measurement at 28 weeks did not significantly differ between the cesarean section and the vaginal delivery groups (*p* = 0.588). In the unadjusted model, women were more likely undergo a successful TOLAC if they were nonobese (*p* < 0.001), had a previous vaginal birth or VBAC (*p* = 0.008), had spontaneous labor (*p* = 0.010), and did not have hypertension in pregnancy (*p* = 0.004) (Table 4).

**Table 4:** Univariable analysis of association between maternal characteristics and TOLAC.

Variables	Vaginal birth (%) *n* = 364, 71.1%	Cesarean (%) *n* = 148, 28.9%	*P*-value
Maternal age (y)			
<35	283 (77.7)	108 (73.0)	0.249
≥35	81 (22.3)	40 (27.0)	
Maternal height (cm)	162.38 ± 5.12	162.47 ± 5.04	0.857
Pre-pregnancy BMI (kg/m2)			
<18.5	34 (9.3)	17 (11.5)	<0.001
18.5–24.9	297 (81.6)	82 (55.4)	
25–29.9	33 (9.1)	49 (33.1)	
Gestational age of weight measured (at 28 weeks)	28.20 ± 0.6	28.23 ± 0.61	0.588
Previous Vaginal birth or VBAC			
Yes	42 (11.5)	6 (4.1)	0.008
No	322 (88.5)	142 (95.9)	
Labor induction			
Yes	60 (16.5)	39 (26.4)	0.010
No	304 (83.5)	109 (73.6)	
Gestational age of delivery			
<40 weeks	243 (66.8)	105 (70.9)	0.357
≥40 weeks	121 (33.2)	43 (29.1)	
Hypertension in pregnancy			
Yes	35 (9.6)	28 (18.9)	0.004
No	329 (90.4)	120 (81.1)	
Diabetes in pregnancy			
Yes	45 (12.4)	15 (10.1)	0.477
No	319 (87.6)	133 (89.9)	

BMI, pre-pregnancy body mass index; TOLAC, trial of labor after cesarean; VBAC, vaginal birth after cesarean section.

After adjusting for pre-pregnancy body mass index, induction of labor, previous vaginal birth or VBAC, hypertension in pregnancy, and other potential confounders, women who gained excessive weight after 28 weeks had significantly lower odds of vaginal birth than those who did not (Adjusted odds ratio [aOR] 0.31, 95% CI 0.20–0.48); however, excessive weight gain before 28 weeks had no significant effect on the VBAC rate (aOR 0.81, 95% CI 0.52–1.23) (Table 5).

**Table 5:** Multivariable logistic regression of GWG and VBAC.

Variables	Vaginal birth (%) *n* = 364	Cesarean (%) *n* = 148	*P*-value	Adjust^†^ OR	95% confidence internal
GWG Before 28 weeks (IOM category)			0.272		
Not excess	245 (67.3)	94 (63.5)		ref	-
Excessive	119 (32.7)	54 (36.5)		0.81	0.52–1.23
GWG After 28 weeks (IOM category)			<0.001		
Not excess	232 (63.7)	47 (31.8)		ref	-
Excessive	132 (36.3)	101 (68.2)		0.31	0.20–0.48

GWG, gestational week gain; IOM, Institute of Medicine. †Adjusted for pre-pregnancy body mass index, induction of labor, hypertension in pregnancy, previous Vaginal birth. All of which were significant at the *p* < 0.05 level with VBAC in univariable analysis.

Table 6 shows the association between GWG after 28 weeks and the VBAC rate stratified by GWG before 28 weeks. We found that among women with excessive or non-excessive GWG before 28 weeks, the non-excessive GWG after 28 weeks group had less risk of failed TOLAC than the excessive GWG after 28 weeks group (aOR 0.27, 95% CI 0.12–0.60; aOR 0.32, 95% CI 0.19–0.55; respectively).

**Table 6:** Associations of GWG after 28 weeks with VBAC stratified by GWG before 28 weeks.

GWG before28weeks (IOM category)	GWG after 28 weeks (IOM category)	Vaginal birth *n* (%)	Cesarean *n* (%)	*p*	Crude OR (95% CI)	Adjust^†^ OR (95% CI)
Not excess	Not excess	33 (35.1)	165 (67.3)	<0.001	ref	ref
	Excessive	61 (64.9)	80 (32.7)		0.26 (0.16–0.43)	0.32 (0.19–0.55)
Excessive	Not excess	14 (25.9)	67 (56.3)	<0.001	ref	ref
	Excessive	40 (74.1)	52 (43.7)		0.27 (0.13–0.55)	0.27 (0.12–0.60)

GWG, gestational week gain; IOM, Institute of Medicine; OR, odds ratio; 95%CI, 95% confidence internal. ^†^Adjusted for pre-pregnancy body mass index, induction of labor, hypertension in pregnancy, previous Vaginal birth.

The aORs from the multivariable logistic regression models stratified by pre-pregnancy BMI are shown in Figure 1. We found that excessive gestational weight gain after 28 weeks was associated with a lower success rate of TOLAC than non-excessive weight gain in underweight, normal weight, and overweight women (aOR 0.23, 95% CI 0.06–0.88; aOR 0.42, 95% CI 0.25–0.70; and aOR 0.12, 95% CI 0.04–0.36; respectively). Gestational weight gain before 28 weeks was not associated with VBAC in any BMI category in this study.

## 4. Discussion

Among Chinese women in this study, excessive GWG after 28 weeks was linked to a higher risk of failed TOLAC among women with or without excessive GWG before 28 weeks. Compared with non-excessive GWG, excessive GWG after 28 weeks also reduces the VBAC rate among underweight, normal, and overweight women.

We found that nearly half of the women gained more weight than recommended after 28 weeks (45.5%), which was higher than that before 28 weeks (33.8%), contrary to the findings of a previous study which showed the GWG between the second and third trimesters was approximately the same in the underweight, normal, and overweight groups (28). Overweight women overweight women had a higher rate of excessive weight gain after 28 weeks of gestation, and other studies have shown that overweight and obese women are more likely to gain too much weight during pregnancy than women with a normal BMI (20, 29). We also revealed that women who exceeded the IOM recommended weight gain after 28 weeks had a higher likelihood of having induced labor and gestational hypertension, which is consistent with previous studies (20, 23). Interestingly, we found that women with diabetes in pregnancy tended not to exceed the IOM recommendations for GWG after 28 weeks. This result is consistent with previous studies, Shi et al. also found that less than the recommended amount of weight gain by the IOM was observed in more than one-third of the pregnant women who had GDM (17). A potential explanation for this finding is that the women with GDM who received the diagnosis between the 24th and 28th week of pregnancy managed to lower their blood glucose levels and curb their excessive weight gain by following a healthy diet and engaging in physical activity (30). Moreover, women who were prone to GDM were more likely to receive education about appropriate weight gain throughout pregnancy during prenatal care, in order to reduce the occurrence of known pregnancy complications, such as preeclampsia and fetal macrosomia. Therefore, these women might pay more attention to their dietary habits, have more physical activity, and ultimately have less GWG than women without GDM (31).

The overall chance of a successful TOLAC was 71.1%. Previous findings found that women who had delivered vaginally had a higher chance of vaginal birth (30). Our study also confirmed most prior reports that abnormal pre-pregnancy weight is linked to an increased rate of failed TOLAC (32, 33). Shi and his colleagues performed a retrospective cohort study that encompassed all TOLAC cases in the US from 2012 to 2020 to determine if overweight women were significantly less likely to undergo TOLAC (32). Herman and his colleagues found a link between maternal BMI and TOLAC utilization in a trial involving 536 pregnant women (33). In addition, consistent with previous studies (34, 35), we found that induction of labor and gestational hypertension were associated with failed TOLAC.

There is limited research on GWG and TOLAC outcomes. The likelihood of successful VBAC was reported to be 40% lower in patients who gained over 40 pounds by Juhasz and his colleagues (20), and Shi Hanxu and his team also found that weight gain exceeding 20 pounds during pregnancy was linked to a lower VBAC rate (32). In contrast, Jenny and her colleagues did not observe any effect of excessive GWG on TOLAC success rates (19). However, these studies did not performed a stratified analysis by pre-pregnancy BMI and gestational stage, which are potential modifiers of the relationship between GWG and TOLAC outcomes. Our results revealed that excessive GWG after 28 weeks was association with failed TOLAC. Further analysis found that increased risk of failed TOLAC was linked to excessive GWG after 28 weeks among women with or without excessive GWG before 28 weeks. Inconsistent with our study findings, IS et al. (24) suggested that trimester-specific GWG was not significantly associated with cesarean delivery in general outside the framework of TOLAC.

Two population-based studies analyzed the association between GWG and cesarean section stratified by pre-pregnancy BMI in pregnant women generally and arrived at different conclusions. Xu and his colleagues conducted a population-based cohort study with 174,953 singleton pregnancies and found that high GWG was associated with a higher overall risk of cesarean delivery for all BMI groups (36). On the other hand, a population-based cohort study which included 245,526 singleton term pregnancies, suggested that high weight gain during pregnancy increased the risk of cesarean delivery for obese women (37). In our study, we found that excessive GWG after 28 weeks was associated with a higher chance of cesarean delivery for all BMI groups (underweight, normal weight, and overweight), whereas the vaginal delivery was not influenced by excessive weight gain before 28 weeks.

The associations between excessive GWG after 28 weeks and the increased risks of failed TOLAC in our study might owing to several mechanisms. Excessive GWG after 28 weeks is positively related to pre-pregnancy overweight, induction of labor, and hypertension in pregnancy, which in turn could contribute to the increased risks of failing TOLAC (20, 23, 29). Moreover, excessive GWG may impair uterine contraction by increasing the amount of fat and cholesterol in the uterine muscle, resulting in reduced uterine contractility, longer labor duration, and more cesarean deliveries (38). However, it is unknown if the distribution of excessive fat differs before and after 28 weeks of gestation. Since fat distribution is an important factor in uterine contraction, whether excessive weight gain in the third trimester is more likely to lead to a relative increase in the distribution of fat in the uterus requires further research.

Some limitations of our study should be acknowledged. First, our study was conducted in a single center and excluded obese women, which may affect the applicability of our findings to other populations. Second, this was a retrospective study, which may lead to selection bias. It is possible that women with a high pre-pregnancy BMI were counseled toward choosing elective repeat cesarean delivery, and this may be one of the reasons why there were so few obese people in this study. Third, due to data limitations, we were unable to perform further analyses based on metabolic-related laboratory indicators (blood glucose, hemoglobin, etc.) and physical examination indicators (abdominal circumference, subcutaneous fat, etc.). Fourth, although our analysis stratified by different pre-conception BMI groups, there may be other unknown confounding variables which may influenced VBAC we did not account for in our analysis. Moreover, we did not further analyze women with gestational diabetes, such as whether exercise would affect their weight. Therefore, more studies are needed to explore this issue in depth in the future.

## 5. Conclusion

To sum up, our findings showed that regardless of pre-pregnancy weight status and weight gain before 28 weeks, excessive weight gain after 28 weeks of pregnancy was associated with decreased rates of VBAC. This finding suggests the importance of weight management in the third trimester to improve the chance of VBAC, especially in overweight women, who were more likely to gain excessive weight after 28 weeks. Our results suggest that women can increase their likelihood of achieving VBAC by managing their weight during pregnancy. Based on our results, we recommend that TOLAC women should be counseled to avoid excessive GWG after 28 weeks, no matter their pre-pregnancy BMI, to increase their possibility of VBAC success.

## Data availability statement

The original contributions presented in the study are included in the article/supplementary material, further inquiries can be directed to the corresponding author.

## Ethics statement

The study was approved by the Ethics Committee of the Fourth affiliated Hospital of Hebei Medical University (2022KS010). The patients/participants provided their written informed consent to participate in this study.

## Author contributions

GL and HS conceived and designed the study and drafted the manuscript. GL, JZ, and HZ collected the clinical data. GL and CZ analyzed the data. All authors contributed to the article and approved the submitted version.

## Funding

This work was supported by the Shanghai Educational Science Research Project (C2023160), Shanghai Clinical Research Center for Gynecological Diseases (22MC1940200), and Shanghai Urogenital System Diseases Research Center (2022ZZ01012).

## Conflict of interest

The authors declare that the research was conducted in the absence of any commercial or financial relationships that could be construed as a potential conflict of interest.

## Publisher’s note

All claims expressed in this article are solely those of the authors and do not necessarily represent those of their affiliated organizations, or those of the publisher, the editors and the reviewers. Any product that may be evaluated in this article, or claim that may be made by its manufacturer, is not guaranteed or endorsed by the publisher.
